# Preparation and Mechanism of Toughened and Flame-Retardant Bio-Based Polylactic Acid Composites

**DOI:** 10.3390/polym15020300

**Published:** 2023-01-06

**Authors:** Kai Xu, Chentao Yan, Chunlin Du, Yue Xu, Bin Li, Lubin Liu

**Affiliations:** 1 Heilongjiang Key Laboratory of Molecular Design and Preparation of Flame Retarded Materials, College of Chemistry, Chemical Engineering and Resource Utilization, Northeast Forestry University, Harbin 150040, China; 2 Key Laboratory of Forest Plant Ecology, Ministry of Education, Northeast Forestry University, Harbin 150040, China; 3 Key Laboratory of Bio-Based Material Science and Technology, Ministry of Education, Northeast Forestry University, Harbin 150040, China

**Keywords:** polylactic acid, toughening, high-effective flame retardant, mechanism

## Abstract

As a biodegradable thermoplastic, polylactic acid (PLA) shows great potential to replace petroleum-based plastics. Nevertheless, the flammability and brittleness of PLA seriously limits its use in emerging applications. This work is focused on simultaneously improving the flame-retardancy and toughness of PLA at a low additive load via a simple strategy. The PLA/MKF/NTPA biocomposites were prepared by incorporating alkali-treated, lightweight, renewable kapok fiber (MKF) and high-efficiency, phosphorus-nitrogenous flame retardant (NTPA) into the PLA matrix based on the extrusion–injection molding method. When the additive loads of MKF and NTPA were 0.5 and 3.0 wt%, respectively, the PLA/MKF/NTPA biocomposites (PLA3.0) achieved a rating of UL-94 V-0 with an LOI value of 28.3%, and its impact strength (4.43 kJ·m^−2^) was improved by 18.8% compared to that of pure PLA. Moreover, the cone calorimetry results confirmed a 9.7% reduction in the average effective heat of combustion (av-EHC) and a 0.5-fold increase in the flame retardancy index (FRI) compared to the neat PLA. NTPA not only exerted a gas-phase flame-retardant role, but also a condensed-phase barrier effect during the combustion process of the PLA/MKF/NTPA biocomposites. Moreover, MKF acted as an energy absorber to enhance the toughness of the PLA/MKF/NTPA biocomposites. This work provides a simple way to prepare PLA biocomposites with excellent flame-retardancy and toughness at a low additive load, which is of great importance for expanding the application range of PLA biocomposites.

## 1. Introduction

As global industrialization continues, people are faced with the problems of fossil resource depletion and the resulting environmental pollution [1]. Consequently, replacing traditional petroleum-based materials with renewable bio-based materials has become a hot topic of current research. Among them, PLA, a thermoplastic material with good biodegradability, biocompatibility, and processability, is an important product in the renewable chemistry industry [2,3]. Specifically, PLA is produced by polymerizing lactic acid or lactide from the fermentation of crops [4,5,6], which has shown great potential for application in emerging fields, such as the auto industry, in electronic devices, and so on [3,7]. However, the flammability and poor toughness of PLA have limited its application in emerging fields [3,8].

Many efforts have been made to enhance the flame-retardancy of PLA [7]. Examples include inorganic flame-retardant fillers [9], intumescent flame retardants (IFR) [10], and phosphorus-nitrogen flame retardants (P-N FRs) [11,12,13,14,15,16,17]. The inorganic flame-retardant fillers commonly used for PLA are metal hydroxides [18], nano-clays [9], layered double hydroxides (LDHs) [19], and expanded graphite (EG) [20]. Among the inorganic flame-retardant fillers, the low-cost, low-toxicity aluminum hydroxide (ATH) often requires a 50–60 wt% addition to give PLA a UL-94 V-0 rating [18]. Meanwhile, IFRs have shown application potential in oxygenated polymers since they were first applied to thermoplastics in the 1980s [21,22,23]. Zhang et al. prepared a novel bio-based IFR by compounding ammonium polyphosphate (APP) and modified lignin [24]. When the additive load of IFR was 23 wt%, the PLA/IFR compound obtained a UL-94 V-0 grade. In summary, high loading amounts of inorganic flame-retardant fillers and IFR were required to achieve a satisfactory flame-retardant rating for PLA, and these levels seriously destroyed the mechanical properties of PLA. Recently, P-N FRs have shown highly efficient flame-retardant PLA composites [12,13,14]. The reason is that the flame-retardant effect of P-N FRs is not only reflected in the promotion of char formation, but also in the capture of free radicals in the gas phase [12,14]. Xue et al. [12] prepared an oligomeric phosphoramidite poly(phenyl phosphate) piperazine (PPP) with a one-pot method. With an addition of 3 wt% PPP, the PLA composites achieved a UL-94 V-0 grade with only an 8.6% reduction in mechanical properties. In addition, our group synthesized a series of high-efficiency P-N FRs of PLA [13,14,15]. When the additions of N,N′,N″-(nitrilotris(ethane-2,1diyl))tris(P,P-diphenylphosphinic amide) (NTPA) were 2.5 wt%, the flame-retardant PLA biocomposites achieved a UL-94 V-0 rating, and the results indicated that phosphoramide flame retardants showed excellent flame-retardancy in PLA [15].

Although the introduction of P-N FRs improved the flame-retardancy of PLA, the brittleness of PLA has not been solved. Polymers [25,26,27,28], core-shell flame retardants [29,30,31,32], and synthetic or natural fiber materials [33,34,35,36,37] are commonly used as toughening modifiers for PLA. For instance, unsaturated polyester and polyurethane have been used to improve the toughness of PLA via reactive blending [26,28]. The toughening effect of synthetic polymers on PLA is remarkable, but the non-degradability of petroleum-based molecules is still not negligible. The introduction of core-shell flame retardants is another strategy to toughen PLA [38,39], as they can form abundant hydrogen bonds with PLA and are beneficial to improve their compatibility with PLA, but the tedious self-assembly process is challenging for large-scale production. Comparatively, biofiber-reinforced plastics have the advantages of degradability, flexibility in fabrication, and being inexpensive [40]. However, there are fewer studies on the flame-retardancy of biofiber-reinforced PLA. Introducing flame retardants such as APP [41,42], diammonium phosphate [43], and nanoclay is a feasible strategy [44]. Recently, Niu et al. [37,45,46] prepared flame-retardant and toughened PLA by compounding hybrid aerogel-coated bamboo fibers with APP. Compared with neat PLA, the prepared aerogel-coated biofiber-reinforced PLA showed a 37–42% increase in toughness, benefiting from its superior interfacial adhesion. As we all know, natural fibers have superior biocompatibility than synthetic fibers, and their introduction hardly changed the biodegradability of PLA itself. Among them, kapok fiber (KF) is seed fiber derived from the Ceiba pentandra tree [47,48], and it has the characteristics of light weight, high specific surface area (97% highest hollow ratio [49]), renewability, and excellent biocompatibility and degradability [47]. The low additive load of hollow and lightweight KF has great potential to improve the brittleness of PLA. Therefore, the integration of KF with P/N flame retardants is a predictable and easy approach to improve the toughness and flame-retardancy of PLA simultaneously.

Herein, the purpose of this work was to simultaneously improve the toughness and flame-retardancy of PLA while maintaining the biodegradation characteristics of PLA composites themselves. Therefore, we introduced alkali-treated, renewable, lightweight KF (MKF) and phosphonamide flame retardants (NTPA) into a PLA matrix through the extrusion–injection molding method. The fire safety, thermal stability, and flame-retardant mechanisms of the PLA/MKF/NTPA biocomposites were systematically studied. In addition, their mechanical properties were also evaluated, and the corresponding toughening mechanism was proposed.

## 2. Materials and Methods

### 2.1. Materials

**Scheme 1:**
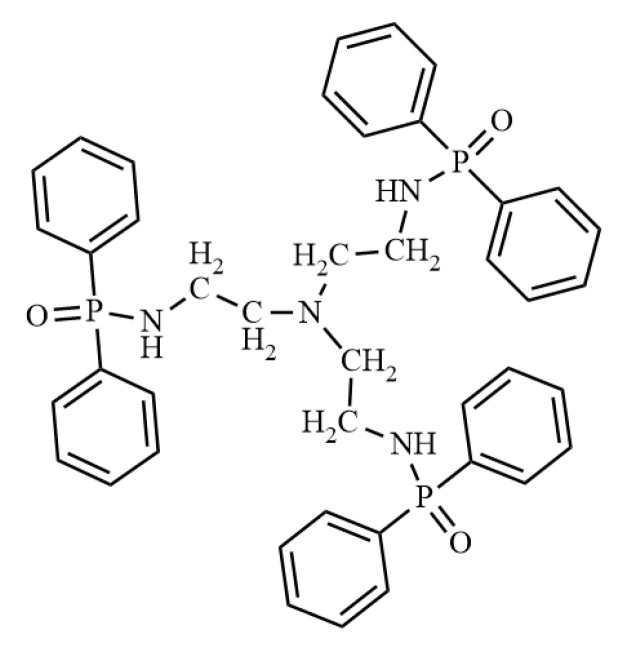
The chemical structure of NTPA.

Kapok fibers (KF) were purchased from Husheng Trading Co., Ltd. (Nanjing, China). NaOH was provided by Fuchen Chemical Reagent Co., Ltd. (Shanghai, China). Polylactic acid (3052D) was obtained from Nature Works Co., Ltd. NTPA was synthesized according to our previous work [15], and its chemical structure is illustrated in Scheme 1.

### 2.2. Preparation of PLA/MKF/NTPA Biocomposites

The original KF (150–200 μm) were pretreated with 0.8 wt% NaOH solution to remove waxes from their surfaces and referred to as modified KF (MKF) after washing and drying. Then, the PLA and MKF were dried (70 °C, 6 h) before use. Subsequently, the PLA, MKF, and NTPA were uniformly blended in a mixer. The mixed sample was granulated with a twin-screw extruder (SLJ-20); the temperature profiles of the twin-screw extruder were 165, 170, 175, 178, 180, and 180 °C during the melt compounding. After the extrusion operation, the composites were cut into pellets. Then, the final test specimens were molded with an HTF86X1 hydraulic injection molding machine (Zhejiang Haitian Equipment Company, China) at the temperature parameters of 165, 170, 175, 178, and 180 °C, and the formulations are given in Table 1.

### 2.3. Characterization

The tensile and flexural strengths of the composites were measured with a Reger computer-controlled RGT-20A mechanical apparatus (Shenzhen, China) at rates of 5 and 2 mm·min^−1^ following the ASTM D638 and D790 standards, respectively. The notched impact strength was performed with an XJC-5 impact tester (Hebei, China) per the ASTM D256 standard.

The limiting oxygen index (LOI) tests were carried out with a JF-3 oxygen index instrument (Jiangning, China) based on the ASTM D2863 standard. The size of the specimens was 130 mm × 6.5 mm × 3.2 mm. The UL-94 tests for the specimens (130 mm × 13 mm × 3.2 mm) were carried out on a CZF-2-type instrument (Jiangning, China) based on the ASTM D3801-1996 standard.

A cone calorimetry instrument (West Sussex, UK) was used to evaluate the combustion performance of samples following the ISO 5660 standards. The samples with a size of 100 mm × 100 mm × 3 mm were measured at the heat flux of 35 kW·m^−2^.

The microscopic morphology characteristics of the specimens were observed with a scanning electron microscopy (SEM) instrument (JSM7500F microscope, Japan).

X-ray photoelectron spectroscopy (XPS) was performed using an ultra-high vacuum system and a Kα hemispherical electron analyzer (Thermofisher Scientific Company, Waltham, MA, USA). The corresponding C, N, O, and P elements were analyzed.

Thermogravimetric coupled with Fourier transform infrared (TG-IR) analysis of the samples was performed with the TGA Q5000 IR thermogravimetric analyzer interfaced with the Nicolet 6700 FTIR spectrophotometer. The 5.0 mg sample was heated under a nitrogen atmosphere from 50 to 800 °C with a flow rate of 20 mL·min^−1^.

## 3. Results and Discussion

### 3.1. Mechanical Property Analysis of PLA/MKF Biocomposites

**Figure 1:**
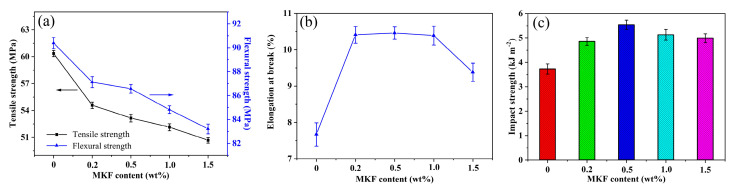
Mechanical properties of PLA/MKF biocomposites. (**a**) Tensile strength and flexural strength, (**b**) elongation at break, and (**c**) notched Izod impact strength.

To identify the toughening role of MKF, the mechanical properties of PLA/MKF biocomposites were characterized. The tensile strength (60.35 MPa), flexural strength (90.39 MPa), elongation at break (7.67%), and notched Izod impact strength (3.73 kJ·m^−2^) of neat PLA are depicted in Figure 1. When the MKF was introduced, the tensile and flexural strength of PLA/MKF biocomposites decreased, whereas their ductility and impact strength increased significantly. When the additive load of MKF increased to 0.5 wt%, the maximum impact strength (5.54 kJ·m^−2^) of the PLA/MKF biocomposites was obtained, which was an increase of 48.5% over neat PLA. Similar results were also reported by Qian et al. [35] and Wang et al. [36]. These researchers observed that the incorporation of fiber-like materials gave rise to an improvement in toughness at the sacrifice of the tensile strength of PLA composites. In comparison with bamboo cellulose nanowhiskers (BCNW) [35], only 0.5 wt% MKF significantly improved the toughness of the PLA/MKF biocomposites, indicating that the lightweight hollow MKF has a clear advantage in giving PLA superior toughness at a low additive load.

### 3.2. Fire Safety Analysis of PLA/MKF/NTPA Biocomposites

From previous studies, it was determined that 0.5 wt% MKF imparted excellent toughness to PLA composites, thus identifying 0.5 wt% as the optimum additive load. As a consequence, the PLA/MKF/NTPA biocomposites were prepared by introducing NTPA to improve their flame-retardant performance.

The flame-retardant effect of NTPA on the PLA/MKF/NTPA biocomposites was investigated, and the related data are illustrated in Table 1. Neat PLA was highly inflammable and burned with a large number of molten drops, which was attributed to the rapid degradation of molecular chains. When NTPA was added in the amount of 3 wt%, the PLA3.0 biocomposite passed the UL-94 V-0 rating with an LOI value of 28.3%. Although NTPA could not completely inhibit the melt dripping of the PLA/MKF/NTPA biocomposites, the droplets from the PLA/MKF/NTPA biocomposites did not ignite the absorbent cotton in the UL-94 test because of the excellent gas-phase flame-retardant effect of NTPA, thus giving the PLA/MKF/NTPA biocomposites good fire safety.

**Figure 2:**
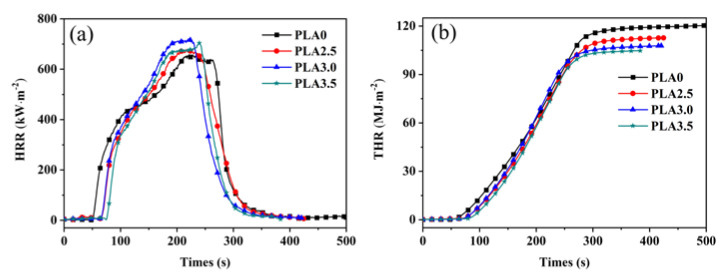
(**a**) HRR and (**b**) THR curves of PLA/MKF/NTPA biocomposites.

The fire safety of the PLA/MKF/NTPA biocomposites was further investigated by simulating the combustion behavior of the composites under fire disaster conditions via cone calorimetry tests [13]. The curves of the heat release rate (HRR) and total heat release (THR) of the biocomposites are depicted in Figure 2. The related data are also presented in Table 2. With the incorporation of NTPA, the time to ignition (TTI) of biocomposites increased from 51 s for PLA0 to 74 s for PLA3.5, indicating that NTPA inhibited the ignition of PLA biocomposites. In Table 2, the peak HRR (pHRR) of PLA0 was 657 kW·m^−2^, which was lower than that of neat PLA (766 kW·m^−2^) in our previous work [15]. This was attributed to the production of a thin char layer by MKF during the combustion of PLA0, which acted as a barrier in the condensed phase. When the flame broke through the barrier layer, the HRR increased rapidly again. Thus, the THR of PLA0 was higher, rising to 120.4 MJ·m^−2^. When NTPA was incorporated, the pHRR of the PLA/MKF/NTPA composites slightly increased. This phenomenon was attributed to the decomposition of NTPA that then promoted the degradation of the bio-based matrix. However, the THR and the average effective heat of combustion (av-EHC) of PLA3.5 were reduced by 13.0% and 12.3%, respectively, compared with those of PLA0. Meanwhile, the total smoke release (TSR) of the PLA biocomposites increased from 4.96 m^−2^·m^−2^ for PLA0 to 268.02 m^−2^·m^−2^ for PLA3.5, which corresponds with the increasing NTPA content. This indicated that the efficient flame-retardant effect of NTPA on PLA biocomposites was mainly due to the gas-phase flame-retardant effect [50]. In addition, Vahabi et al. reported a general dimensionless criterion FRI (Flame Retardant Index, Equation (1)) that was used to evaluate the flame-retardancy of thermoplastic materials [51].

(1) $$FRI=\frac{{[THR\;\times (pHRR/TTI)]}_{PLA}}{{[THR\;\times (pHRR/TTI)]}_{PLA\;composites}}$$

As shown in Table 2, the FRI of the composites improved with the addition of NTPA, and the FRI of PLA3.5 was 1.55 times more than that of PLA0. The results indicated that PLA/MKF obtained better flame retardancy with the introduction of NTPA.

### 3.3. Thermal Stability of PLA/MKF/NTPA Biocomposites

**Figure 3:**
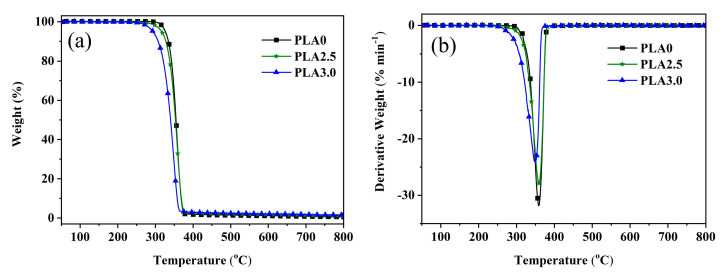
(**a**) TG and (**b**) DTG curves of PLA/MKF/NTPA biocomposites under N_2_.

The TG and DTG curves of the PLA/MKF/NTPA biocomposites are presented in Figure 3 under N_2_. The corresponding data are summarized in Table 3. As with PLA0, there was only one decomposition process for all PLA composites. However, the T_initial_ (the 5 wt% degradation temperature) reduced from 326.5 °C for PLA0 to 316.6 and 293.4 °C for PLA2.5 and PLA3.0, respectively, due to the catalytic degradation effect of NTPA. Meanwhile, the T_max_ (maximum degradation temperature) of the PLA3.0 biocomposites shifted forward with increasing NTPA level, and their R_max_ (weight loss rate at T_max_) lowered from 31.8%·min^−1^ for PLA0 to 24.1%·min^−1^. This demonstrated that NTPA efficiently lowered the thermal decomposition rate of the PLA/MKF/NTPA biocomposites. In addition, the char residue at 800 °C of the PLA3.0 biocomposites increased from 0.48% for PLA0 to 1.44%. These results demonstrated that the introduction of NTPA was beneficial for PLA/MKF to obtain flame-retardancy.

### 3.4. Flame-Retardant Mechanism of PLA/NTPA/MKF Biocomposites

**Figure 4:**
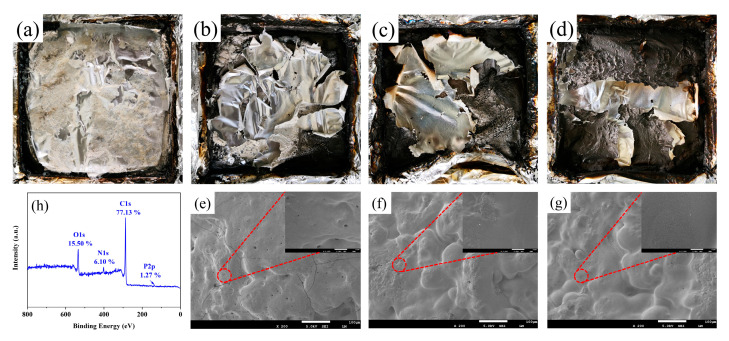
Digital images of the residual char of (**a**) PLA0, (**b**) PLA2.5, (**c**) PLA3.0, and (**d**) PLA3.5 after CCT; SEM images (200× and 2000×) of the residual char of (**e**) PLA2.5, (**f**) PLA3.0, and (**g**) PLA3.5 after CCT; (**h**) XPS analysis of char residues of PLA3.0 after CCT.

According to the combustion mode of different flame-retardant materials, the flame-retardant mechanism is classified into condensed and gas-phase mechanisms [23]. The condensed-phase flame-retardant mechanism of the PLA/MKF/NTPA biocomposites was analyzed by examining photos, SEM images, and the elemental composition of char residues after the CCT. The relevant images and data are shown in Figure 4. PLA0 burned completely without any residual char (Figure 4a). When NTPA was introduced, the residual char of the PLA/MKF/NTPA biocomposites increased and gradually formed a nearly complete char layer (Figure 4b–d). The microscopic morphology of the PLA/MKF/NTPA biocomposites’ char residue was continuous, and the pores and cracks on the surface of the residue gradually decreased with increasing NTPA content, which indicated the catalytic carbonization effect of NTPA on the biocomposites. Specifically, compared with PLA2.5 (Figure 4e), the cracks and pores of residual char for PLA3.0 (Figure 4f) disappeared gradually, and a compact and denser char formed, which effectively prevented the internal and external mass and heat during the combustion of the PLA/MKF/NTPA biocomposites. As a result, PLA 3.0 obtained a satisfactory UL-94 V-0 rating. In Figure 4h, XPS analysis indicated that the elemental contents of C, N, O, and P in the residual char of PLA3.0 were 77.13, 6.10, 15.50, and 1.27 wt%, respectively. The N and P elements in the char residue further supported the catalytic charring effect of NTPA [15].

**Figure 5:**
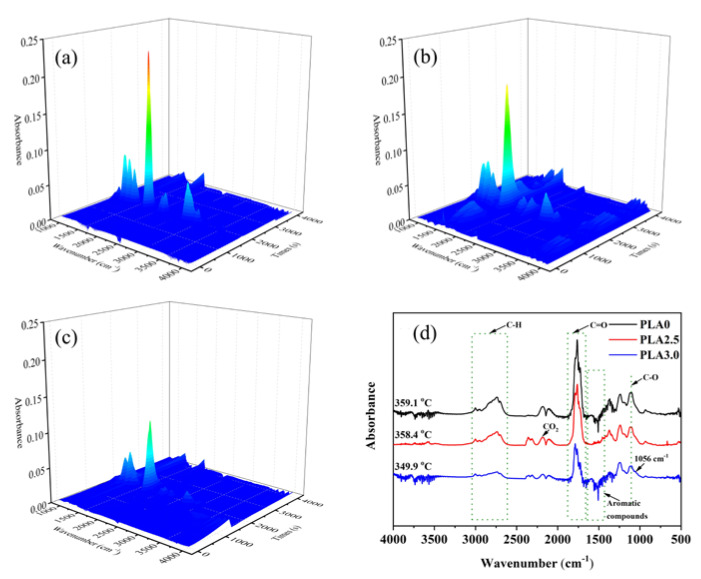
Three-dimensional TG-FTIR spectra of pyrolysis products of (**a**) PLA0, (**b**) PLA2.5, and (**c**) PLA3.0; (**d**) FTIR spectra at T_max_ of PLA/MKF/NTPA biocomposites.

The gas-phase flame-retardant mechanism of the PLA/MKF/NTPA composites was studied with TG-IR analysis, and the relevant data are shown in Figure 5. The 3D TG-FTIR spectra (Figure 5a–c) of the PLA/MKF/NTPA biocomposites suggested that the IR absorption intensity of the biocomposites diminished with the improvement of NTPA content, which indicated that NTPA reduced the total amount of gas-phase thermal decomposition products of the PLA/MKF/NTPA composites. The FTIR spectra at T_max_ of the biocomposites were compared in Figure 5d. With the introduction of NTPA, the intensity of the absorption peaks of alkanes (C-H), carbonyls (C=O), and ethers (C-O-C) [52,53] in the pyrolysis products of PLA/MKF/NTPA biocomposites decreased significantly, and the absorption peaks of aromatic compounds and PO· appeared at 1586, 1483, and 1056 cm^−1^ [15]. The interesting results indicated that NTPA changed the pyrolysis path of the composites and that the benzene- and phosphorus-containing radicals generated by NTPA decomposition acted as quenchers in the gas phase.

The flame-retardant effect of NTPA on the composites was found in the condensed phase as well as in the gas phase. The flame-retardant effect of NTPA in the PLA/MKF/NTPA biocomposites was dominated by the gas-phase flame-retardant mechanism and supplemented by the condensed-phase flame-retardant mechanism.

### 3.5. Mechanical Properties and Toughening Mechanism of PLA/MKF/NTPA Biocomposites

**Figure 6:**
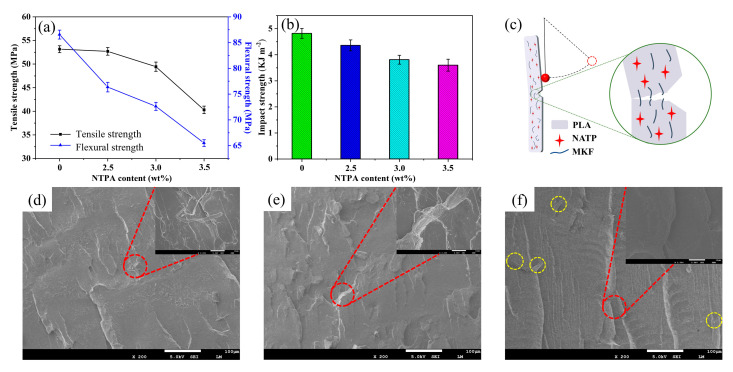
Mechanical properties of PLA/MKF/NTPA biocomposites. (**a**) Tensile strength and flexural strength, (**b**) notched Izod impact strength. (**c**) Energy absorption effect of MKF in impact tests. SEM images (200× and 2000×) of the fracture surfaces of (**d**) PLA0, (**e**) PLA2.5, and (**f**) PLA3.0.

The effect of NTPA on the mechanical properties of PLA/MKF/NTPA was evaluated, and the corresponding data is shown in Figure 6. The tensile strength, flexural strength, and notched Izod impact strength of the PLA/MKF/NTPA biocomposites exhibited a decreasing trend with increasing NTPA levels. This was because the rigid NTPA molecules presented a certain plasticizing effect in the PLA/MKF/NTPA biocomposites, thus resulting in a slight reduction of mechanical properties. However, compared to neat PLA (3.73 kJ·m^−2^, Figure 1), the impact strength of PLA2.5 (4.86 kJ·m^−2^) and PLA3.0 (4.43 kJ·m^−2^) still increased by 30.3% and 18.8%, respectively. Specifically, PLA3.0 achieved a UL-94 V-0 rating and showed momentous application potential in flame-retardant and toughened PLA biocomposites.

The fracture surface morphology of the PLA/MKF/NTPA biocomposites was characterized by SEM, as shown in Figure 6d–f. The fracture surface of the PLA/MKF/NTPA biocomposites became significantly rougher compared to that of neat PLA [15]. Furthermore, a fiber-like structure was observed on the fracture surfaces of the biocomposites, which was attributed to the fracture of the filled MKF. These fracture surface morphologies indicated that NTPA and MKF were homogeneously dispersed and compatible in the PLA matrix, and that there was no obvious agglomeration of NTPA and MKF. The corresponding toughening mechanism is elucidated in Figure 6c. During the impact process, MKF acted as an energy absorber to dissipate stress and enhance the impact strength of the PLA/MKF/NTPA biocomposites.

**Figure 7:**
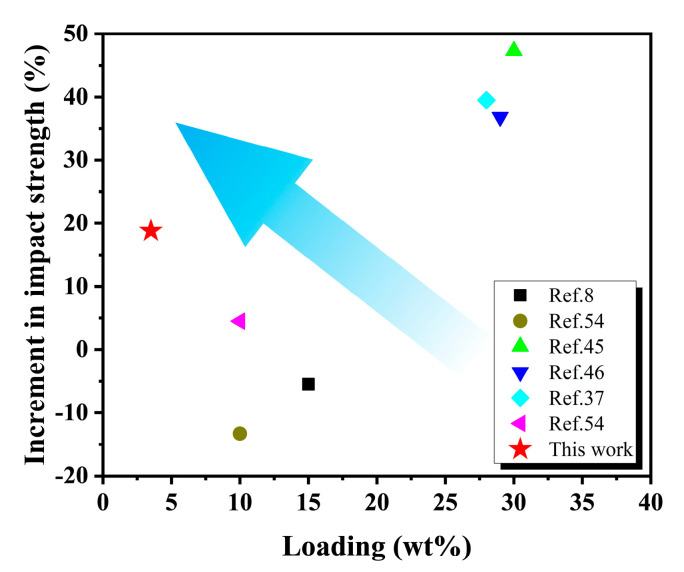
Comparisons of variation in impact strength and loading of PLA3.0 with previously reported flame-retardant and toughened PLA materials containing biofiber which achieved a UL-94 V-0 rating.

To clarify the flame-retardancy and toughening levels of the prepared PLA/MKF/NTPA biocomposites, filler loadings and impact strength increments were used to compare with previously reported PLA composites containing biofiber materials. The comparison results are shown in Table 4 and Figure 7. It should be noted that the impact strength increments were derived by comparison with neat PLA. In general, low additive loading means low cost, whereas high loading often causes deterioration of tensile properties due to poor dispersion. Compared with previously reported PLA composites [8,37,45,46,54], the PLA/MKF/NTPA biocomposites exhibited excellent flame-retardancy and toughness at low additive loads (only 3.5 wt%), and they thus showed potential applications in large-scale production.

## 4. Conclusions

In this work, the flame-retardant and toughened PLA biocomposites were prepared successfully by introducing low additive loads of bio-based MKF and NTPA. Only a 3.0 wt% additive load of NTPA enabled the PLA/MKF/NTPA biocomposites to pass the UL-94 V-0 rating, and the LOI value of the PLA/MKF/NTPA biocomposites increased from 19.2% to 28.3%. In addition, the av-EHC of the PLA3.0 biocomposites declined by 9.8%, and their char residue increased significantly. The excellent fire safety of the PLA biocomposites was attributed to the gas-phase quenching effect of the phosphorus- and benzene-containing radicals of NTPA as well as the barrier effect of char residues induced by NTPA. In addition, the homogenously dispersed lightweight MKF enabled the impact strength of the PLA/MKF/NTPA biocomposites to obtain an 18.8% increase over neat PLA. This work presented a simple, low-cost way for the preparation of toughened and flame-retardant PLA biocomposites at low additive loads, and it has great potential for emerging fields.

## Figures and Tables

**Table 1:** Formulation and the UL-94 and LOI test results of the PLA/MKF/NTPA biocomposites.

Sample	Components (wt%)			PLA Biocomposites			
	PLA	NTPA	MKF	t_1_/t_2_ (s)	UL-94	LOI (%)	Dripping/Ignition of Cotton
PLA0	99.5	0	0.5	10.16/92.16	Fail	19.2	Yes/Yes
PLA2.5	97.0	2.5	0.5	9.09/2.18	V-1	27.2	Yes/No
PLA3.0	96.5	3.0	0.5	2.31/0.72	V-0	28.3	Yes/No
PLA3.5	96.0	3.5	0.5	1.11/0.62	V-0	29.6	Yes/No

**Table 2:** CCT data of PLA/MKF/NTPA biocomposites.

Samples	TTI (s)	pHRR (kW·m^−2^)	T_PHRR_ (s)	THR (MJ·m^−2^)	av-EHC (MJ·kg^−1^)	TSR (m^−2^·m^−2^)	FRI
PLA0	51	657.1	222	120.4	26.64	4.96	1.00
PLA2.5	67	678.5	217	112.7	25.12	42.43	1.36
PLA3.0	67	715.0	218	107.8	24.03	125.51	1.35
PLA3.5	74	705.4	239	104.7	23.36	268.02	1.55

**Table 3:** Thermal degradation data of PLA/MKF/NTPA biocomposites.

Sample	T_initial_ (°C)	R_max_/T_max_ (%·min^−1^/°C)	Char Residue (wt%, 800 °C)
PLA0	326.5	31.8/359.1	0.48
PLA2.5	316.6	27.8/358.4	0.84
PLA3.0	293.4	24.1/349.9	1.44

**Table 4:** The detailed comparison with previously reported flame-retardant and toughened PLA materials containing biofiber.

Fillers	Loading (wt%)	UL-94	Increment in Impact Strength (%)	Reference
PN-FR@CNF	15	V-0	−5.5	[8]
APP + MCC	7 + 3	V-0	−13.3	[54]
APP + IBF-Si	10 + 20	V-0	47.3	[45]
APP + MBF	9 + 20	V-0	36.8	[46]
APP + BF-SiP	8 + 20	V-0	39.5	[37]
APP + MA-MCC	7 + 3	V-0	4.5	[54]
NTPA + MKF	3 + 0.5	V-0	18.8	This work

PN-FR@CNF: core shell nanofibrous flame-retardant system; APP + MCC: ammonium polyphosphate + microcrystalline cellulose; IBF-Si: silicon aerogel-modified bamboo fiber; MBF: hybrid aerogel containing silica-nitrogen-modified bamboo fiber; BF-SiP: hybrid aerogel containing silica-phosphorus-modified bamboo fiber; MA-MCC: methacrylic acid microcrystalline cellulose.

## Data Availability

The data presented in this study are available on request from the corresponding authors.

## References

[1] Iwata T. (2015). Biodegradable and Bio-Based Polymers: Future Prospects of Eco-Friendly Plastics. Angew. Chem. Int. Ed..

[2] Zhu Y., Romain C., Williams C.K. (2016). Sustainable Polymers from Renewable Resources. Nature.

[3] Castro-Aguirre E. (2016). Poly(Lactic Acid)—Mass Production, Processing, Industrial Applications, and End of Life. Adv. Drug Deliv. Rev..

[4] Wang X., Wang S., Wang W., Li H., Liu X., Gu X., Bourbigot S., Wang Z., Sun J., Zhang S. (2020). The Flammability and Mechanical Properties of Poly (Lactic Acid) Composites Containing Ni-MOF Nanosheets with Polyhydroxy Groups. Compos. Part B Eng..

[5] Râpă M., Miteluţ A.C., Tănase E.E., Grosu E., Popescu P., Popa M.E., Rosnes J.T., Sivertsvik M., Darie-Niţă R.N., Vasile C. (2016). Influence of Chitosan on Mechanical, Thermal, Barrier and Antimicrobial Properties of PLA-Biocomposites for Food Packaging. Compos. Part B Eng..

[6] Arif Z.U., Khalid M.Y., Noroozi R., Sadeghianmaryan A., Jalalvand M., Hossain M. (2022). Recent Advances in 3D-Printed Polylactide and Polycaprolactone-Based Biomaterials for Tissue Engineering Applications. Int. J. Biol. Macromol..

[7] Tawiah B., Yu B., Fei B. (2018). Advances in Flame Retardant Poly(Lactic Acid). Polymers.

[8] Feng J., Sun Y., Song P., Lei W., Wu Q., Liu L., Yu Y., Wang H. (2017). Fire-Resistant, Strong, and Green Polymer Nanocomposites Based on Poly(Lactic Acid) and Core–Shell Nanofibrous Flame Retardants. ACS Sustain. Chem. Eng..

[9] Morgan A.B. (2006). Flame Retarded Polymer Layered Silicate Nanocomposites: A Review of Commercial and Open Literature Systems. Polym. Adv. Technol..

[10] Feng C., Liang M., Jiang J., Huang J., Liu H. (2016). Flame Retardant Properties and Mechanism of an Efficient Intumescent Flame Retardant PLA Composites. Polym. Adv. Technol..

[11] Liao F., Ju Y., Dai X., Cao Y., Li J., Wang X. (2015). A Novel Efficient Polymeric Flame Retardant for Poly (Lactic Acid) (PLA): Synthesis and Its Effects on Flame Retardancy and Crystallization of PLA. Polym. Degrad. Stab..

[12] Xue Y., Shen M., Zheng Y., Tao W., Han Y., Li W., Song P., Wang H. (2020). One-Pot Scalable Fabrication of an Oligomeric Phosphoramide towards High-Performance Flame Retardant Polylactic Acid with a Submicron-Grained Structure. Compos. Part B Eng..

[13] Xu Y., Qiu Y., Yan C., Liu L., Xu M. (2021). A Novel and Multifunctional Flame Retardant Nucleating Agent towards Superior Fire Safety and Crystallization Properties for Biodegradable Poly (Lactic Acid). Adv. Powder.

[14] Liu L., Xu Y., Di Y., Xu M., Pan Y., Li B. (2020). Simultaneously Enhancing the Fire Retardancy and Crystallization Rate of Biodegradable Polylactic Acid with Piperazine-1,4-Diylbis(Diphenylphosphine Oxide). Compos. Part B Eng..

[15] Liu L., Xu Y., Pan Y., Xu M., Di Y., Li B. (2021). Facile Synthesis of an Efficient Phosphonamide Flame Retardant for Simultaneous Enhancement of Fire Safety and Crystallization Rate of Poly (Lactic Acid). Chem. Eng. J..

[16] Liu L., Xu Y., Xu M., Li Z., Hu Y., Li B. (2019). Economical and Facile Synthesis of a Highly Efficient Flame Retardant for Simultaneous Improvement of Fire Retardancy, Smoke Suppression and Moisture Resistance of Epoxy Resins. Compos. Part B Eng..

[17] Xu Y., Zhang W., Qiu Y., Xu M., Li B., Liu L. (2022). Preparation and Mechanism Study of a High Efficiency Bio-Based Flame Retardant for Simultaneously Enhancing Flame Retardancy, Toughness and Crystallization Rate of Poly (Lactic Acid). Compos. Part B Eng..

[18] Woo Y., Cho D. (2013). Effect of Aluminum Trihydroxide on Flame Retardancy and Dynamic Mechanical and Tensile Properties of Kenaf/Poly(Lactic Acid) Green Composites. Adv. Compos. Mater..

[19] Wang D.-Y., Leuteritz A., Wang Y.-Z., Wagenknecht U., Heinrich G. (2010). Preparation and Burning Behaviors of Flame Retarding Biodegradable Poly(Lactic Acid) Nanocomposite Based on Zinc Aluminum Layered Double Hydroxide. Polym. Degrad. Stab..

[20] Fukushima K., Murariu M., Camino G., Dubois P. (2010). Effect of Expanded Graphite/Layered-Silicate Clay on Thermal, Mechanical and Fire Retardant Properties of Poly(Lactic Acid). Polym. Degrad. Stab..

[21] Camino G., Costa L., Trossarelli L. (1984). Study of the Mechanism of Intumescence in Fire Retardant Polymers: Part I—Thermal Degradation of Ammonium Polyphosphate-Pentaerythritol Mixtures. Polym. Degrad. Stab..

[22] Camino G., Costa L., Trossarelli L. (1984). Study of the Mechanism of Intumescence in Fire Retardant Polymers: Part III—Effect of Urea on the Ammonium Polyphosphate-Pentaerythritol System. Polym. Degrad. Stab..

[23] Liu B., Zhao H., Wang Y. (2022). Advanced Flame-retardant Methods for Polymeric Materials. Adv. Mater..

[24] Zhang R., Xiao X., Tai Q., Huang H., Hu Y. (2012). Modification of Lignin and Its Application as Char Agent in Intumescent Flame-Retardant Poly(Lactic Acid). Polym. Eng. Sci..

[25] Wang X., Peng S., Chen H., Yu X., Zhao X. (2019). Mechanical Properties, Rheological Behaviors, and Phase Morphologies of High-Toughness PLA/PBAT Blends by in-Situ Reactive Compatibilization. Compos. Part B Eng..

[26] Li D.-F., Zhao X., Jia Y.-W., He L., Wang X.-L., Wang Y.-Z. (2019). Simultaneously Enhance Both the Flame Retardancy and Toughness of Polylactic Acid by the Cooperation of Intumescent Flame Retardant and Bio-Based Unsaturated Polyester. Polym. Degrad. Stab..

[27] Ma M., Wang X., Liu K., Chen S., Shi Y., He H., Wang X. (2022). Achieving Simultaneously Toughening and Flame-Retardant Modification of Poly(Lactic Acid) by in-Situ Formed Cross-Linked Polyurethane and Reactive Blending with Ammonium Polyphosphate. J. Mater. Sci..

[28] Yang R., Gu G., Tang C., Miao Z., Cao H., Zou G., Li J. (2022). Super-Tough and Flame-Retardant Poly(Lactic Acid) Materials Using a Phosphorus-Containing Malic Acid-Based Copolyester by Reactive Blending. Polym. Degrad. Stab..

[29] Wu G., Liu S., Wu X., Ding X. (2017). Core-Shell Structure of Carbon Nanotube Nanocapsules Reinforced Poly(Lactic Acid) Composites. J. Appl. Polym. Sci..

[30] Yu Y., Zhang Y., Xi L., Zhao Z., Huo S., Huang G., Fang Z., Song P. (2022). Interface Nanoengineering of a Core-Shell Structured Biobased Fire Retardant for Fire-Retarding Polylactide with Enhanced Toughness and UV Protection. J. Clean. Prod..

[31] Zhang Y., Xiong Z., Ge H., Ni L., Zhang T., Huo S., Song P., Fang Z. (2020). Core–Shell Bioderived Flame Retardants Based on Chitosan/Alginate Coated Ammonia Polyphosphate for Enhancing Flame Retardancy of Polylactic Acid. ACS Sustain. Chem. Eng..

[32] Dong X., Wu Z., Wang Y., Li T., Yuan H., Zhang X., Ma P., Chen M., Huang J., Dong W. (2022). Improving the Toughness and Flame Retardancy of Poly (Lactic Acid) with Phosphorus-Containing Core-Shell Particles. J. Appl. Polym. Sci..

[33] Xu H., Liu C.-Y., Chen C., Hsiao B.S., Zhong G.-J., Li Z.-M. (2012). Easy Alignment and Effective Nucleation Activity of Ramie Fibers in Injection-Molded Poly(Lactic Acid) Biocomposites. Biopolymers.

[34] Tan L., Liu L., Liu C., Wang W. (2021). Improvement in the Performance of the Polylactic Acid Composites by Using Deep Eutectic Solvent Treated Pulp Fiber. J. Renew. Mater..

[35] Qian S., Sheng K. (2017). PLA Toughened by Bamboo Cellulose Nanowhiskers: Role of Silane Compatibilization on the PLA Bionanocomposite Properties. Compos. Sci. Technol..

[36] Wang Y., Mei Y., Wang Q., Wei W., Huang F., Li Y., Li J., Zhou Z. (2019). Improved Fracture Toughness and Ductility of PLA Composites by Incorporating a Small Amount of Surface-Modified Helical Carbon Nanotubes. Compos. Part B Eng..

[37] Niu Q., Yue X., Guo Z., Yan H., Fang Z., Li J. (2022). Flame Retardant Bamboo Fiber Reinforced Polylactic Acid Composites Regulated by Interfacial Phosphorus-Silicon Aerogel. Polymer.

[38] Liu L., Yao M., Zhang H., Zhang Y., Feng J., Fang Z., Song P. (2022). Aqueous Self-Assembly of Bio-Based Flame Retardants for Fire-Retardant, Smoke-Suppressive, and Toughened Polylactic Acid. ACS Sustain. Chem. Eng..

[39] Han D., Wang H., Lu T., Cao L., Dai Y., Cao H., Yu X. (2022). Scalable Manufacturing Green Core–Shell Structure Flame Retardant, with Enhanced Mechanical and Flame-Retardant Performances of Polylactic Acid. J. Polym. Environ..

[40] Yiga V.A., Lubwama M., Pagel S., Benz J., Olupot P.W., Bonten C. (2021). Flame Retardancy and Thermal Stability of Agricultural Residue Fiber-Reinforced Polylactic Acid: A Review. Polym. Compos..

[41] Shukor F., Hassan A., Islam S., Mokhtar M., Hasan M. (2014). Effect of Ammonium Polyphosphate on Flame Retardancy, Thermal Stability and Mechanical Properties of Alkali Treated Kenaf Fiber Filled PLA Biocomposites. Mater. Des..

[42] Shumao L., Jie R., Hua Y., Tao Y., Weizhong Y. (2010). Influence of Ammonium Polyphosphate on the Flame Retardancy and Mechanical Properties of Ramie Fiber-Reinforced Poly(Lactic Acid) Biocomposites. Polym. Int..

[43] Suardana N.P.G., Ku M.S., Lim J.K. (2011). Effects of Diammonium Phosphate on the Flammability and Mechanical Properties of Bio-Composites. Mater. Des..

[44] Hapuarachchi T.D., Peijs T. (2010). Multiwalled Carbon Nanotubes and Sepiolite Nanoclays as Flame Retardants for Polylactide and Its Natural Fibre Reinforced Composites. Compos. Part A Appl. Sci. Manuf..

[45] Niu Q., Yue X., Guo Z., Fang Z., Li J. (2022). Strengthening and Flame Retarding Effect of Bamboo Fiber Modified by Silica Aerogel on Polylactic Acid Composites. Constr. Build. Mater..

[46] Niu Q., Yue X., Cao W., Guo Z., Fang Z., Chen P., Li J. (2022). Interfacial Silicon-nitrogen Aerogel Raise Flame Retardancy of Bamboo Fiber Reinforced Polylactic Acid Composites. Int. J. Biol. Macromol..

[47] Zheng Y., Wang J., Wang A. (2021). Recent Advances in the Potential Applications of Hollow Kapok Fiber-Based Functional Materials. Cellulose.

[48] Zhang X., Shi H., Liu L., Min C., Liang S., Xu Z. (2022). Construction of MoS2/Mxene Heterostructure on Stress-Modulated Kapok Fiber for High-Rate Sodium-Ion Batteries. J. Colloid Interface Sci..

[49] Cao Y., Xie L., Sun G., Su F., Kong Q.-Q., Li F., Ma W., Shi J., Jiang D., Lu C. (2018). Hollow Carbon Microtubes from Kapok Fiber: Structural Evolution and Energy Storage Performance. Sustain. Energy Fuels.

[50] Wu N., Fu G., Yang Y., Xia M., Yun H., Wang Q. (2019). Fire Safety Enhancement of a Highly Efficient Flame Retardant Poly (Phenylphosphoryl Phenylenediamine) in Biodegradable Poly(Lactic Acid). J. Hazard. Mater..

[51] Vahabi H., Kandola B., Saeb M. (2019). Flame Retardancy Index for Thermoplastic Composites. Polymers.

[52] Swinehart D.F. (1962). The Beer-Lambert Law. J. Chem. Educ..

[53] Xu K., Tian X., Cao Y., He Y., Xia Y., Quan F. (2021). Suppression of Smoldering of Calcium Alginate Flame-Retardant Paper by Flame-Retardant Polyamide-66. Polymers.

[54] Zhu T., Guo J., Fei B., Feng Z., Gu X., Li H., Sun J., Zhang S. (2020). Preparation of Methacrylic Acid Modified Microcrystalline Cellulose and Their Applications in Polylactic Acid: Flame Retardancy, Mechanical Properties, Thermal Stability and Crystallization Behavior. Cellulose.

